# Full‐length coding sequence analysis of the voltage‐gated sodium channel and acetylcholinesterase genes reveals target‐site mutations and acetylcholinesterase gene duplication in housefly (*Musca domestica*) populations in Japanese livestock barns

**DOI:** 10.1111/mve.70048

**Published:** 2026-01-14

**Authors:** Mikie Nakagawa, Kentaro Itokawa, Nozomi Uemura, Yasuhiro Tomioka, Tsutomu Tanikawa, Kyo Itoyama, Shinji Kasai, Osamu Komagata

**Affiliations:** ^1^ Department of Agriculture Meiji University Kanagawa Japan; ^2^ Department of Medical Entomology Japan Institute for Health Security Tokyo Japan; ^3^ Ikari Shodoku Co., Ltd. Chiba Japan

**Keywords:** acetylcholinesterase, carbamate, haplotype, insecticide resistance, *kdr*, livestock barn, *Musca domestica*, organophosphate, pyrethroid, voltage‐gated sodium channel

## Abstract

Houseflies (*Musca domestica* L.), a widespread pest in livestock facilities, have developed resistance to commonly utilized insecticides, including pyrethroids, organophosphates and carbamates. However, the genetic status of insecticide resistance in Japanese housefly populations remains largely elusive. We used hybridization probe capture and next‐generation sequencing (NGS) to examine the mutations across the entire coding sequence of two insecticide target genes, voltage‐gated sodium channel (VGSC) and acetylcholinesterase (AChE), from 240 individual *M. domestica* collected from 14 livestock facilities in Japan between 2013 and 2024. This study is the first to utilize these molecular methods to examine insecticide resistance in houseflies collected from the field. Resistance‐related mutations in *VGSC* (e.g., *super‐kdr* [M918T + L1014F], *kdr‐His* [L1014H] and L1014F) were detected in most wild populations, with differing allele frequencies. Notably, *super‐kdr* was widespread (12/15 populations) with frequencies ranging from 0.06 to 1.00, whereas *kdr‐His* was observed in 8/15 populations with 0.03–0.70 frequencies. For *AChE*, three known resistance mutations (V260L, G342A/V and F407Y) were observed in all populations, with at least one resistance allele present at 0.65–1.00 frequencies. An elevation in NGS read counts for *AChE* was noted in several populations, along with the presence of three distinct alleles at amino acid position 342, suggesting AChE gene duplication. These duplications may help in mitigating the fitness costs linked to AChE resistance. Our results show the widespread presence and complexity of insecticide resistance mutations in Japanese *M. domestica* populations and underscore the use of NGS‐based probe capture techniques for monitoring the evolution of resistance.

## Graphical abstract

- We simultaneously analysed insecticide resistance mutations in *VGSC* and *AChE* using NGS and hybridization probe capture in houseflies collected from livestock facilities for the first time.
- Resistance mutations in both *VGSC* and *AChE* were detected in most wild populations, suggesting widespread distribution of resistance genes.
- *AChE* gene duplication was suggested, which may help mitigate fitness costs associated with resistance‐related amino acid substitutions.

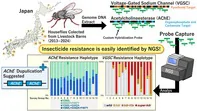

## INTRODUCTION

The housefly, *Musca* is an insect pest commonly found in livestock facilities. It is regarded as a mechanical vector that poses health risks to both humans and livestock because *M. domestica* can carry multiple pathogenic microorganisms on its legs and mouth. *Escherichia coli* O157 and *Campylobacter* are human pathogens that are potentially transmitted by *M. domestica* (Khamesipour et al., 2018). Additionally, *M. domestica* is suspected to be involved in the transmission of significant infectious diseases in livestock, such as the highly pathogenic avian influenza, which can cause economic damage to the livestock industry (Khamesipour et al., 2018). Particularly, poultry houses provide a favourable environment for the breeding of *M. domestica*. The emergence of a large fly population causes stress to workers and animals in poultry facilities, as well as increases the risk of disease transmission (Khan et al., 2012).

Three main classes of insecticides are commonly used to control adult houseflies in Japanese livestock facilities: pyrethroids, organophosphates (OPs) and carbamates. Pyrethroids act on voltage‐gated sodium channels (VGSCs) situated along the nerve cell axons. They bind to VGSCs, preventing their inactivation and prolonging their channel opening, which disrupts normal nerve signal transmission. Specific amino acid substitutions in VGSCs decrease the interaction between the channel and pyrethroids. These mutations are collectively called knockdown resistance (*kdr*), and selective pressure from pyrethroid usage can increase the *kdr* allele frequency within populations (Wiliamson et al., 1996). Numerous *kdr* variants have been reported in *M. domestica* globally (Roca‐Acevedo et al., 2023). The most prevalent substitution is L1014F (Roca‐Acevedo et al., 2023), in which the leucine at the 1014th amino acid residue is replaced by phenylalanine. The co‐occurrence of M918T and L1014F in the same channel is called *super‐kdr* substitution, which improves resistance (Hyeock Lee et al., 1999). Furthermore, another substitution at the 1014th residue, L1014H (*kdr‐His*), is commonly observed in *M. domestica* in the United States and confers a comparatively lower resistance level than L1014F (Sun et al., 2016).

Contrastingly, OP and carbamate insecticides target acetylcholinesterase (AChE). Several amino acid substitutions in AChE are linked to resistance, among which V260L, G342A/V and F407Y are commonly observed in *M. domestica* across different regions of the world (Adib et al., 2023; You et al., 2025). Multiple substitutions are commonly observed in individual flies (Mertz et al., 2023).

In Japan, extensive research on insecticide resistance in *M. domestica* was conducted between the 1950s and the 1990s. Susceptibility tests had confirmed the presence of resistance to pyrethroids, OPs and carbamates by 1986 (Funaki & Motoyama, 1986). However, no studies have reported the genetic basis of *M. domestica*'s insecticide resistance in livestock facilities in Japan since the 2000s. This study aimed to clarify the presence, frequency and geographical distribution of *VGSC* and *AChE* alleles associated with insecticide resistance in *M. domestica* collected from livestock farms in Japan. Traditionally, detection of insecticide resistance‐associated mutations in pests, including *M. domestica*, has primarily relied on polymerase chain reaction (PCR) and direct sequencing. In 2019, Itokawa et al. applied a combination of hybridization probe capture and next‐generation sequencing (NGS) to explore resistance‐associated mutations in mosquitoes, including *Aedes* and *Culex* species. This approach enables lower cost per base and higher throughput. It also allows genotyping of the entire coding regions of the target sites and identification of all amino acid substitutions, including known resistance mutations. NGS approaches remain in the early stages of adoption, and the present study represents the first application of this method to *M. domestica*.

## MATERIALS AND METHODS

### Houseflies

From 2013 to 2024, *M. domestica* larvae were collected from the manure in 14 livestock barns across Japan and reared to adulthood under laboratory conditions for each population (Table 1 and Figure 1). Populations collected between 2013 and 2021 were maintained for several generations or years under controlled indoor rearing conditions. Table 1 details the specific rearing durations. The flies were reared as described in previous reports (2012) (Ogawa et al., 2012).

**TABLE 1:** Collection details and rearing duration of houseflies sampled from livestock barns (2013–2024).

ID	Collection date	Group	Host animal	Total samples	% females	VGSC (*n*)	AChE (*n*)
1	2013/10/11^a^	Hokkaido	Pig	16	63	16	16
2	2015/9/24^b^	Fukushima	Poultry	16	56	15	16
3	2017/9/14^c^	Kagawa	Poultry	16	31	13	14
4	2021/11/25^d^	Ibaraki A	Poultry	16	63	14	13
5	2022/11/18	Kagoshima	Poultry	27	67	18	12
6	2022/7/28	Nagano	Poultry	8	75	8	7
7	2022/8/22	Oita	Cow	16	69	16	13
8	2023/6/26	Fukuoka	Poultry	16	81	15	13
9	2023/6/19	Ibaraki B	Poultry	16	69	16	13
10	2023/8/25	Tochigi	Cow	16	63	16	16
11	2023/10/27	Ibaraki C	Poultry	16	44	16	9
12	2023/11/7	Chiba 2023^e^	Poultry	16	75	14	14
13	2024/3/22	Miyazaki A	Pig	16	63	16	14
14	2024/4/4	Miyazaki B	Pig	13	69	13	10
15	2024/11/25	Chiba 2024^e^	Poultry	16	38	15	15

^a^ Rearing duration: 8 years.

^b^ Rearing duration: 6 years.

^c^ Rearing duration: 4 years.

^d^ Sampled from F2 (lab‐reared).

^e^ Collected in the same barn.

**FIGURE 1:**
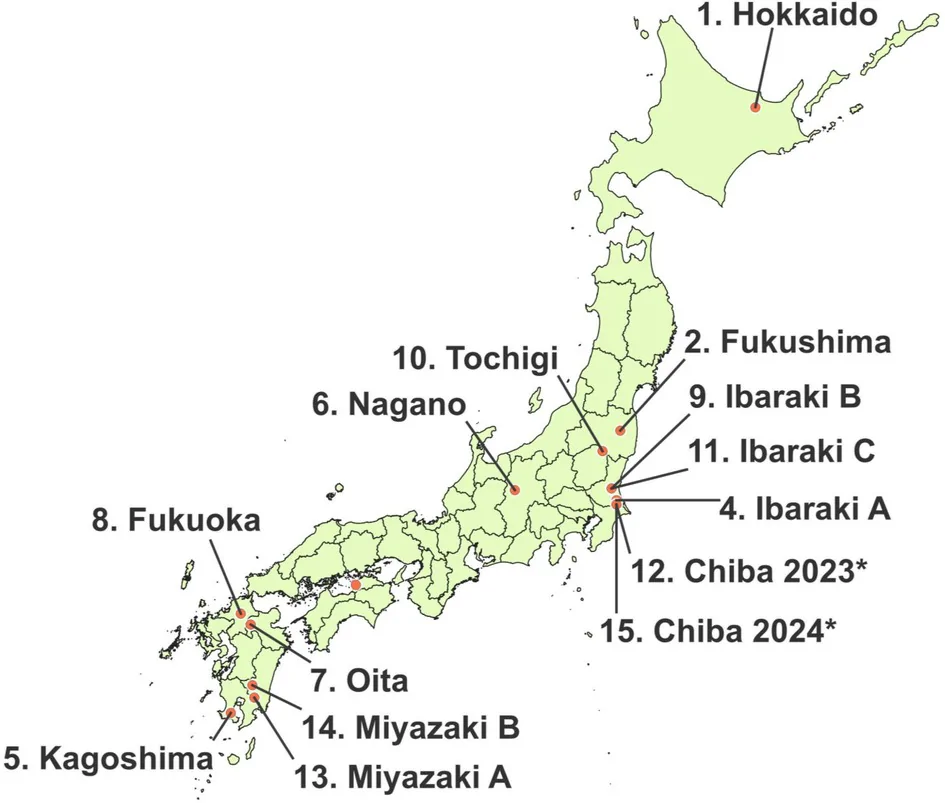
Map of the distribution of houseflies collected in livestock barns between 2013 and 2024. Numbers correspond to those provided in Table 1.

In Chiba Prefecture, to investigate the temporal variation in resistance mutations, samples were collected from the same poultry house over two years. Populations collected in 2023 and 2024 were treated as separate groups (‘Chiba 2023’ and ‘Chiba 2024’).

We used a susceptible reference strain (SRS), a widely recognized insecticide‐susceptible laboratory strain provided by the World Health Organization (WHO) that has been maintained under laboratory conditions for many years (Franco, 1964) as a reference susceptible strain for statistical comparisons. This strain was reared and analysed under the same conditions and with the same methods as the other populations examined in this study.

### Genomic DNA extraction

Genomic DNA was extracted using a Quick‐DNA/RNA™ MagBead Kit (Zymo Research Corporation, California, USA). Homogenization was conducted on the abdomens of individual flies separated using tweezers. Both males and females were randomly selected for the analysis. The extracted DNA was quantified and examined using the QuantiFluor® dsDNA System (Promega, Wisconsin, USA). Extracted DNA was quantified by fluorescence using a Synergy LX microplate reader (Agilent Technologies). DNA samples with concentrations >10 ng/μL were diluted with Milli‐Q water until their concentrations were <10 ng/μL.

### Library preparation

NGS libraries were prepared using the NEBNext® Ultra™ 2 FS DNA PCR‐free Library Prep Kit for Illumina® (New England Biolabs, Massachusetts, USA) at one‐quarter scale of the protocol. Index adapters from Integrated DNA Technologies (IDT) for Illumina DNA/RNA UD indices (Illumina, Inc., California, USA) were utilized.

### Design of the custom hybridization probes

A custom probe set targeting the *VGSC* (GenBank accession: NM_001286885) of *M. domestica* and *Stomoxys calcitrans* L. (Diptera: Muscidae) was designed using the insecticide‐susceptible mutant laboratory strain aabys (BioProject accession: PRJNA1072234) and *Stomoxys calcitrans* (BioProject accession: PRJNA188117 ) as the reference sequences (File S1). Probes of 120‐nt in length were designed to tile all coding exons (CDSs) with approximately 60‐nt overlapping each other. Probes targeting *AChE* were designed based on the genome assembly available in GenBank (BioProject: PRJNA176013) (File S1). All biotinylated oligonucleotides were synthesized using the oPool DNA oligo synthesis service provided by IDT.

### Target enrichment via probe capture

Target sequence enrichment via probe capture was conducted using the xGen™ Hybridization Capture of DNA Libraries kit (IDT, Iowa, USA). After hybridization, PCR amplification was conducted using a HiFi™ PCR Kit (KAPA, Cape Town, South Africa). Libraries were quantified and sequenced in 150 bp paired‐end mode using the MiniSeq Mid Output Kit (300 cycles) on a MiniSeq system (Illumina).

The NGS libraries were quantified using the PrimeTime® Gene Expression Master Mix (Integrated DNA Technologies, IDT) (Itokawa, 2020). For the standard curve, 10 nM PhiX DNA library included in the NextSeq PhiX Control Kit (Illumina, Inc.) was serially diluted to final concentrations of 100, 10, 1.0 and 0.1 pM in Low TE buffer containing 1 ng/μL salmon sperm DNA (Thermo Fisher Scientific, Waltham, USA). Quantitative PCR was performed using the QuantStudio™ 1 Real‐Time PCR System (Thermo Fisher Scientific).

### Bioinformatics analysis of the NGS data

For the raw reads obtained from the NGS, files with sizes <100 × 1024 bytes (100 KB) were excluded from the analysis. Genotyping and phasing were conducted using Strobealign v0.14.0 (Sahlin, 2022), Samtools v1.19.2 (Li et al., 2009), Freebayes v1.3.8 (Garrison & Marth) and Beagle v4.1 (Browning et al., 2021). The downstream statistical analyses excluded samples with insufficient quality, such as those with low on‐target rates after alignment. The criteria for removal based on on‐target rates were set according to the gene (15.0% for VGSC, 5.0% and >500 on_target_reads for AChE).

We used a combination of Bedtools map (v2.30.0) and Samtools depth with the ‘‐a’ option on BAM files to obtain the minimum and median coverage for each CDS region to calculate the gene‐level coverage. Furthermore, we normalized the coverage values by multiplying them by a factor of (5,000,000/total read length) for each sample to enable comparisons across samples with different sequencing depths. This value (5,000,000) was chosen for computational convenience as a round number with no specific biological significance. This normalization effectively standardized all samples to an equivalent of 5 million mapped bases, removing bias owing to variations in library size or sequencing depth. The data were visualized using Matplotlib (v3.7.1).

The relative copy number of the *AChE* was analysed using Bedtools coverage (v2.30.0) to count the number of reads overlapping the CDS regions. We calculated the Reads Per Kilobase (RPK) by normalizing the read counts for *AChE* and *VGSC* based on the resulting read count data. We subsequently calculated the relative RPK as *AChE*/*VGSC* for each sample. This approach uses *VGSC*, which exists as a single copy in the housefly genome (Ingles et al., 1996), as a reference to estimate *AChE* copy number. Welch's *t*‐test was used for statistical comparison between each wild population and the susceptible SRS strain (BioProject accession: PRJNA1264703, BioSample accession: SAMN48577883–SAMN48577886). Data were visualized using Matplotlib (v3.7.1).

### Validation of NGS results by PCR and sanger sequencing

To verify the accuracy of NGS‐based variant detection, direct sequencing by PCR and Sanger methods was performed using a subset of the same individuals (Hokkaido, Fukuoka and Tochigi) analysed by NGS. The *VGSC* regions encompassing amino acid positions 918 and 1014 were amplified and sequenced. Primers were used as described in Kasai et al. (2016). Sequencing was outsourced to Azenta Life Sciences (Tokyo, Japan). The obtained chromatograms were analysed using ATGC software version 14.

Details of DNA extraction methods, PCR reagents and reaction conditions are provided in the Supporting information, together with allele detection results.

## RESULTS

### Voltage‐gated sodium channel

High‐quality sequencing coverage was achieved across all exons of the *VGSC* for all individuals that passed quality filtering (Figure 2). This comprehensive coverage facilitated detection of the pyrethroid resistance‐associated alleles in all 11 housefly populations collected from the livestock barns (Figure 3, File S1). Among these resistance mutations, the *super‐kdr* mutation was identified in 10 populations, *kdr‐His* in seven populations and L1014F in six populations. The total frequency of the resistance alleles differed among the populations, ranging from 0.22 to 1.00.

**FIGURE 2:**
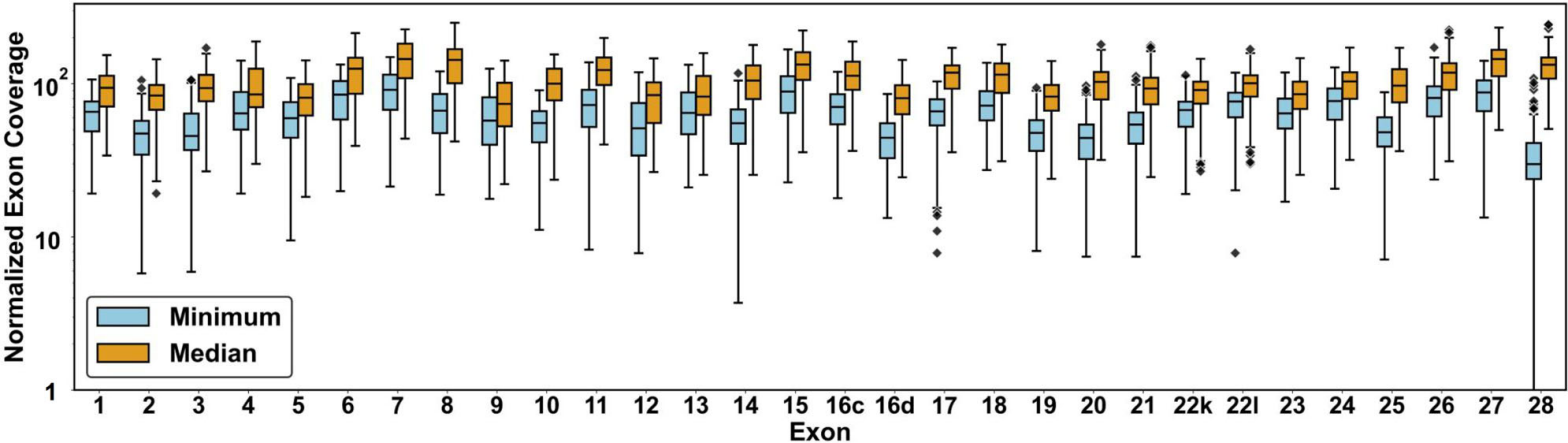
Normalized exon coverage of the voltage‐gated sodium channel gene. Boxplots show the normalized read coverage across voltage‐gated sodium channel exons from the next‐generation sequencing (NGS) data. Coverage values were normalized to 5 million mapped bases per sample as described in Materials and methods section. The blue and orange boxes indicate the minimum and median coverage values, respectively. Exons are sequentially numbered, with alternative exons indicated using letters (16c and 16d).

**FIGURE 3:**
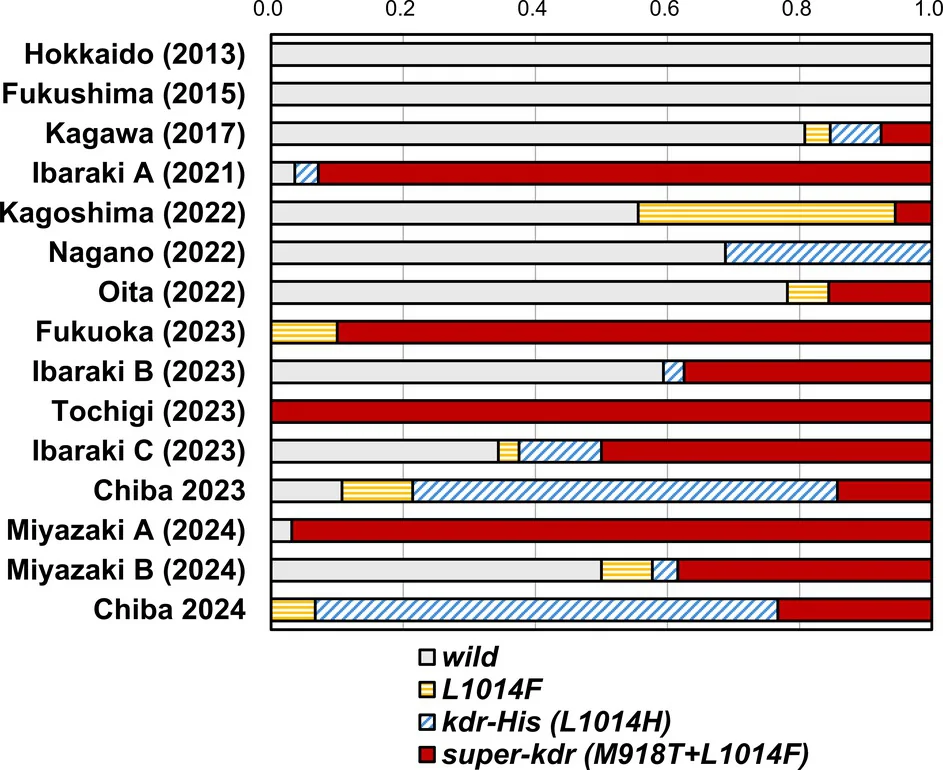
Haplotype frequencies of pyrethroid resistance alleles in houseflies from livestock barns. The stacked bar graph indicates the frequencies of four haplotypes linked to pyrethroid resistance in the voltage‐gated sodium channel gene: *wild‐type* (grey), L1014F (orange horizontal stripes), *kdr‐His* (blue diagonal stripes) and *super‐kdr* (red). Each bar indicates a different collection site. Parentheses indicate the years in which the houseflies were collected.

Contrastingly, different resistance profiles were observed in the three populations that had been reared under laboratory conditions for several years. No pyrethroid resistance mutations were detected in the Fukushima and Hokkaido populations after six and eight years of laboratory rearing, respectively. Contrastingly, in the Kagawa population, which had been maintained for four years, all three mutations—*super‐kdr* (M918T + L1014F), *kdr‐His* (L1014H) and L1014F—were detected, with a combined frequency of 0.2.

Examination of the geographical distribution of the resistance mutations showed distinct regional patterns. All three populations from Ibaraki Prefecture harboured *super‐kdr* and *kdr‐His* mutations at different frequencies. The Ibaraki A population showed the highest *super‐kdr* frequency (0.93), whereas the geographically proximal Ibaraki B and Ibaraki C populations exhibited considerably lower frequencies (0.38 and 0.50, respectively). For *kdr‐His*, the frequencies were more consistent across the Ibaraki population (ranging from 0.07 to 0.31). Contrastingly, the Nagano population from central Japan showed only *kdr‐His* at a frequency of 0.31, and no *super‐kdr* was observed. The Chiba populations, sampled at the same location over two consecutive years, revealed a notable shift: the wild‐type allele was present at a 0.11 frequency in 2023 but was not noted in 2024.

Furthermore, a novel M918V substitution in exon 16c (mutually exclusive of exon 16d) was observed in a heterozygous individual from the Chiba 2023 population (Figure S1) of the heterozygous genotype.

Direct sequencing was performed using a subset of the same individuals analysed by NGS to validate the accuracy of the NGS results. Except for one sample that could not be measured due to low DNA concentration, the genotypes obtained by direct sequencing were fully concordant with those obtained by NGS (File S1).

Additionally, the polymorphisms detected in the SRS were restricted to Exon 28, while Exons 1–27 were in complete agreement with the reference sequence. The detailed polymorphism data for the SRS were omitted from Figure S1 to emphasize the broad variation profile of the wild populations in this study.

### Acetylcholinesterase

High‐quality sequencing coverage was achieved across all *AChE* exons for all individuals that passed quality filtering (Figure 4). Four previously reported resistance‐associated amino acid substitutions, V260L, G342A, G342V and F407Y, were detected in all analysed populations (Figure 5a–c, File S1). These AChE resistance mutations were present at high frequencies (0.5–1.0) in all populations, with all examined flies carrying at least one of these mutations. Notably, they persisted in the Hokkaido and Fukushima colonies despite kdr substitutions being absent in these laboratory‐maintained populations (Figure S2). This indicated that *AChE* resistance mutations persisted under laboratory conditions in which *VGSC* resistance mutations were absent.

**FIGURE 4:**
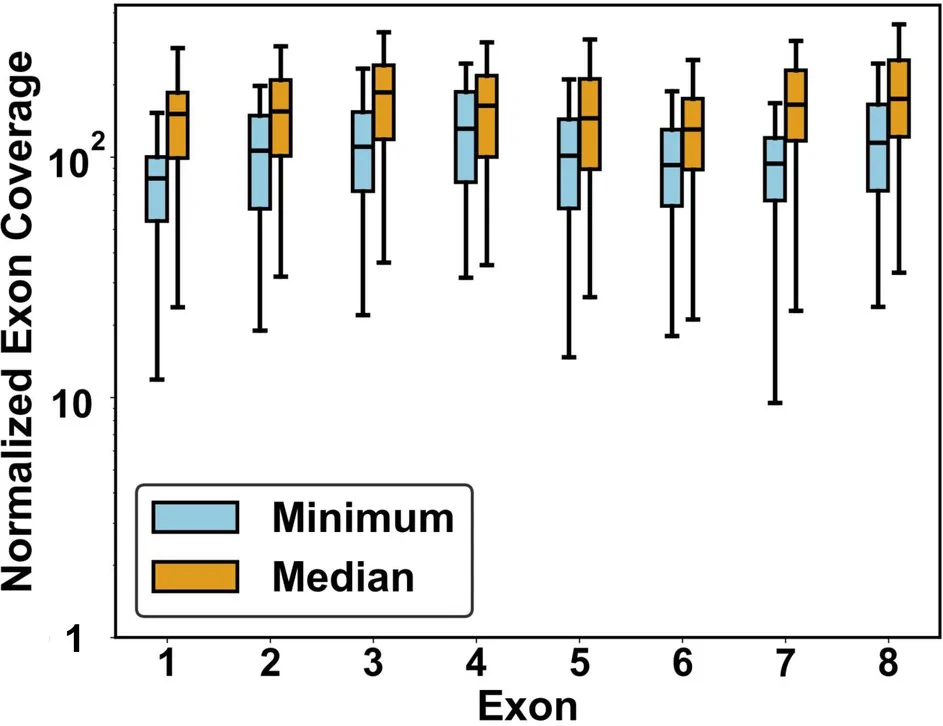
Normalized exon coverage of the acetylcholinesterase gene. Boxplots indicating the normalized read coverage across the exons of the acetylcholinesterase gene from the next‐generation sequencing (NGS) data. Coverage values were normalized to 5 million mapped bases per sample as described in Materials and methods section. The blue and orange boxes represent the minimum and median coverage values, respectively. Exons are sequentially numbered.

**FIGURE 5:**
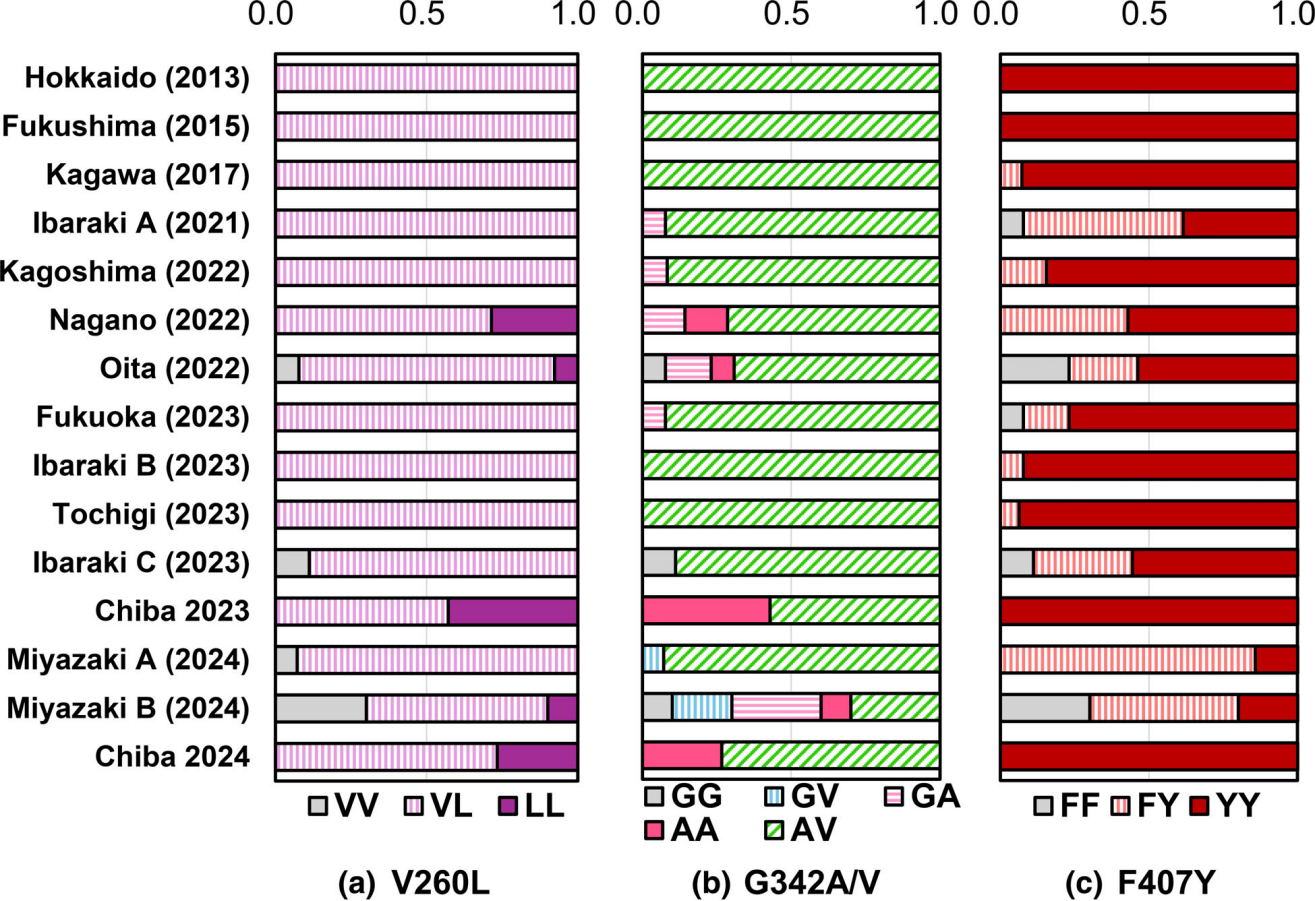
Genotype frequencies of the acetylcholinesterase (AChE) resistance mutations in houseflies from livestock barns. Stacked bar graphs indicate the genotype frequencies of three acetylcholinesterase gene mutations: (a) V260L, (b) G342A/V and (c) F407Y. Each population is represented by a bar with genotypes indicated as 100% stacked proportions.

For the V260L mutation, several individuals carried the variant in a heterozygous state with the wild‐type allele. The V260L genotype frequencies demonstrated considerable deviations from the Hardy–Weinberg equilibrium (HWE) across multiple sampling locations. Several of these deviations reached statistical significance (Table S1). The G342 position exhibited a different pattern. Heterozygous individuals carrying A‐ and V‐variant alleles were frequently observed. Contrastingly, heterozygotes between G342V and the wild‐type G allele were rare, detected in only two populations from Miyazaki Prefecture, at low frequencies (0.07, 0.20). Sequencing analysis showed that some individuals possessed all three alleles (G, A and V) at position 342 (Figure S3). Of the 240 individual houseflies analysed, 29 (12.1%) demonstrated three various alleles at position 342, providing direct gene duplication evidence. Figure S3 shows the detailed allele ratios of these individuals. Figure 6 illustrates the allele composition at amino acid position 342 in three representative individuals from the Ibaraki C (sample number: 7), Kagawa (13) and Chiba 2023 (12) populations. The left panel illustrates the detected ratios of the G:C:T bases (1:1:1, 1:2:1 and 0:2:1), whereas the right panel presents the schematic chromosomal arrangements that explain these observed ratios. A wild‐type (susceptible) strain illustrating normal allelic arrangement without resistance mutations was included as a reference.

**FIGURE 6:**
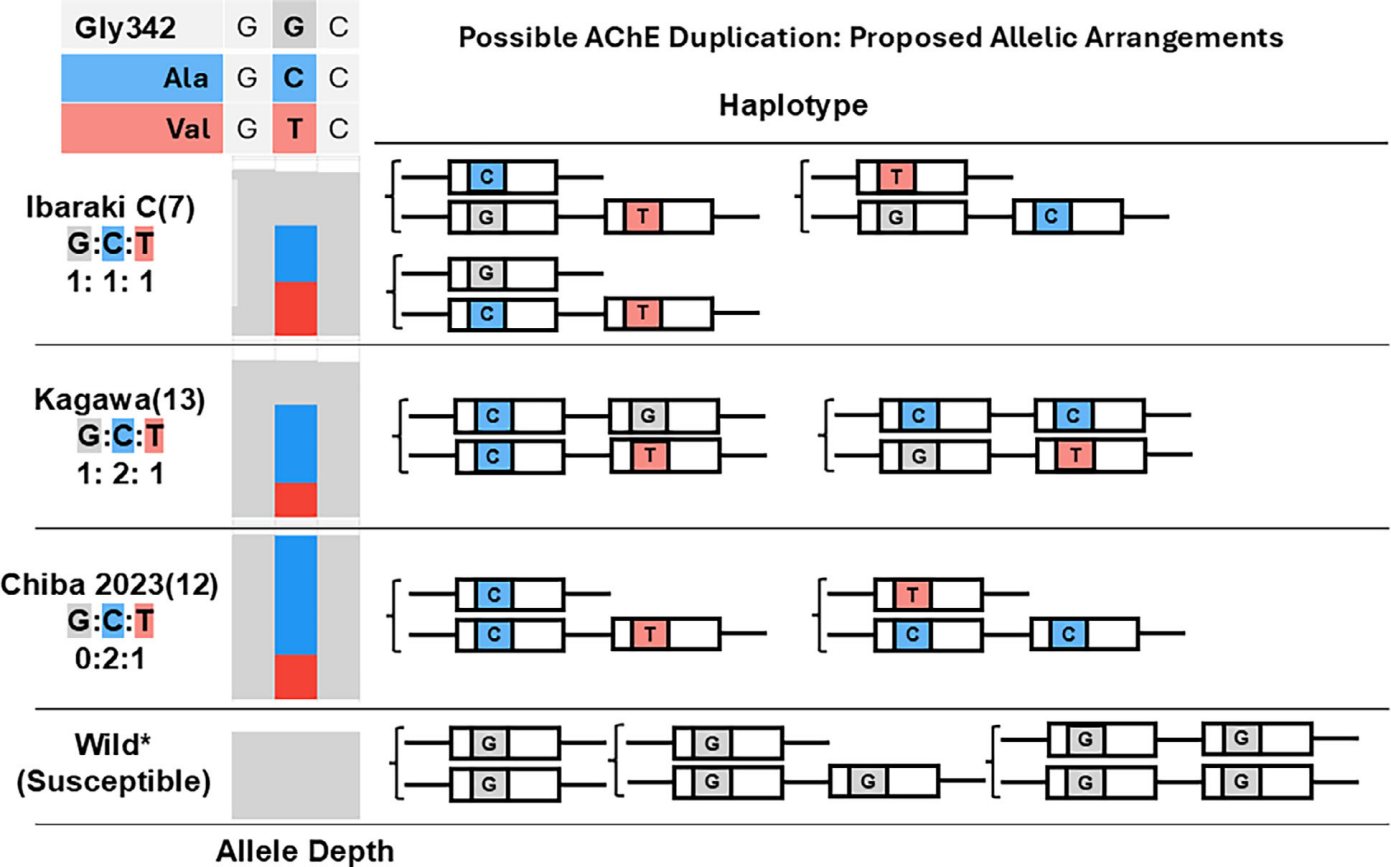
Possible acetylcholinesterase (AChE) gene duplication: proposed allelic arrangements based on the next‐generation sequencing (NGS) allele depth ratios. Each row indicates the allele composition at amino acid position 342, with population and specimen number indicated. Left panel displays the detected allele proportions; right panel shows the proposed chromosomal arrangements explaining the observed ratios. *NGS analysis cannot distinguish between identical sequence copies, potentially underestimating the duplications of identical alleles.

In the SRS, polymorphisms were limited to P11S in Exon 1 and T666A in Exon 9, with all other regions being in complete agreement with the reference sequence. To emphasize the broad mutation profile of the wild populations investigated in this study, the detailed polymorphism data for the SRS were omitted from Figure S2.

### Relative copy numbers of AChE

Figure 7 shows the relative copy numbers of *AChE*, calculated as the ratio of RPKs of the AChE gene to the VGSC gene. Several wild *M. domestica* populations exhibited higher relative *AChE* copy numbers than the SRS. Statistical analysis using Welch's *t*‐test showed that nine populations (Chiba 2023, Chiba 2024, Fukuoka, Hokkaido, Ibaraki B, Ibaraki C, Kagawa, Miyazaki A and Tochigi) showed significantly higher relative *AChE* copy numbers than the SRS (*p* < 0.001). The remaining populations (Fukushima, Ibaraki A, Kagoshima, Miyazaki B, Nagano and Oita) exhibited no statistically significant differences in the relative *AChE* copy numbers compared to the reference strain.

**FIGURE 7:**
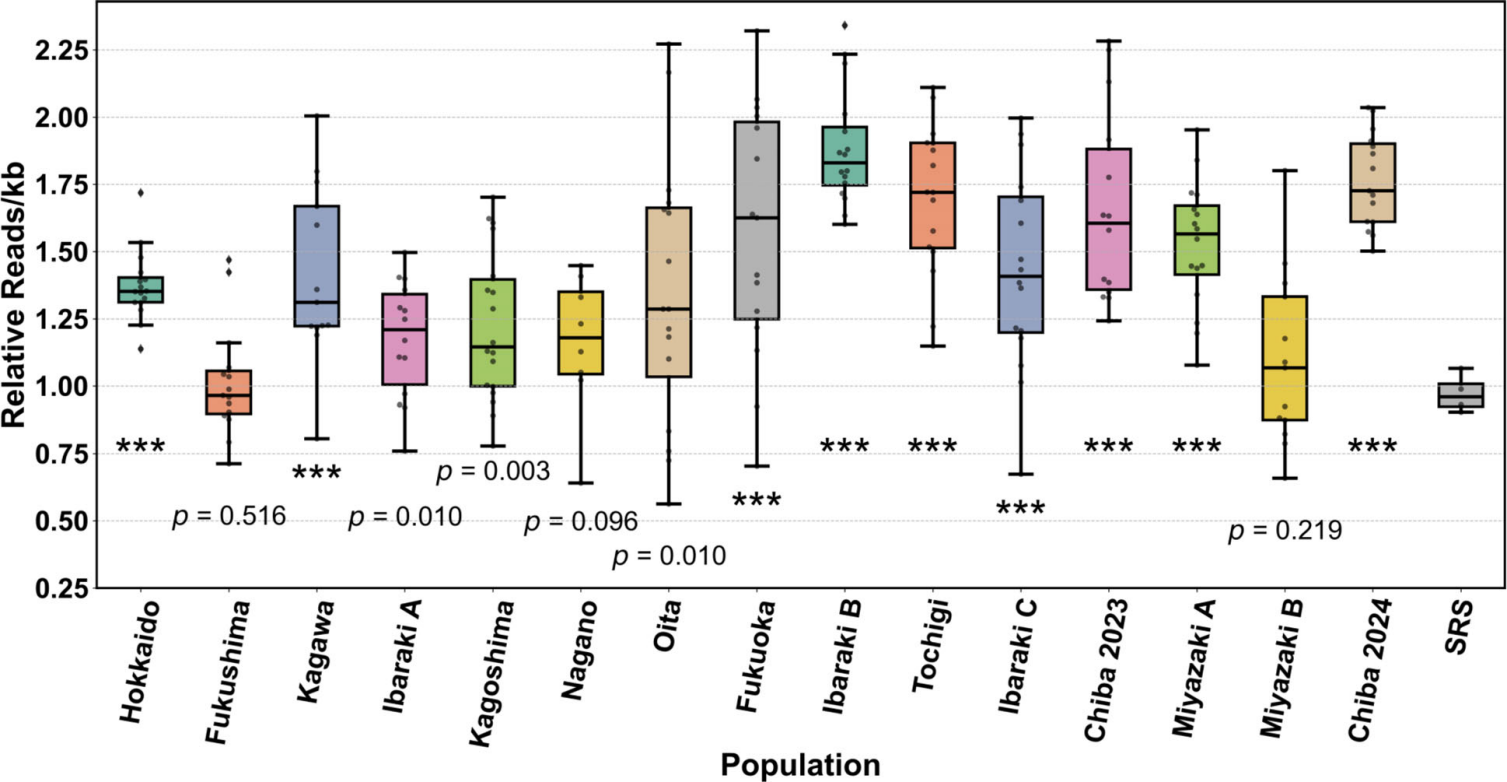
Relative copy numbers of the acetylcholinesterase (AChE) gene compared to the voltage‐gated sodium channel (VGSC) gene. Boxplots show the *AChE*/*VGSC* ratio (Reads Per Kilobase) across housefly populations. This ratio serves as an index of the relative *AChE* gene copy number, with VGSC serving as a single‐copy reference gene. Statistical significance from Welch's *t*‐test comparing each population to susceptible reference strain (SRS) is indicated (***p < 0.001).

## DISCUSSION

Herein, we used the capture probe method, a powerful approach for efficiently analysing the entire coding sequence of insecticide resistance genes (Itokawa et al., 2019), to comprehensively analyse insecticide resistance in houseflies collected from livestock barns. We applied this technique to houseflies collected from livestock barns, enabling simultaneous analysis of all base substitutions in the VGSC and AChE genes. Our comprehensive genomic approach showed three key findings: a widespread distribution of pyrethroid resistance‐associated *VGSC* mutations with distinct regional patterns, a high prevalence of OP/carbamate resistance‐associated *AChE* mutations across all populations, and the first genomic evidence of AChE gene duplication, which may mitigate the fitness costs related to resistance alleles. These results provide substantial insight into the current status and evolutionary mechanisms of insecticide resistance in Japanese housefly populations.

This study reveals the distribution of insecticide resistance mutations in housefly populations in Japan. Three major pyrethroid resistance‐related amino acid substitutions were identified in the VGSC gene. These mutations were mainly found at two critical loci: L1014 and M918. The most prevalent mutation was *super‐kdr*, which combines L1014F and M918T and was detected in 12 of 15 populations with frequencies ranging from 0.06 to 1.00 (Figure 3). These findings indicate the widespread distribution of high‐level pyrethroid resistance across Japanese livestock facilities. Recent reports indicate that the maximum frequency of *super‐kdr* reported in the United States was 0.56, which is lower than the highest frequency observed in this study in Japan (Freeman et al., 2019). *Super‐kdr* was detected in a YPER strain collected in Japan in 1997, indicating that this resistance mutation has been present in Japan for decades (Shono et al., 2002).

L1014H (*kdr‐His*) was detected in 8 out of the 15 populations, with frequencies ranging from 0.03 to 0.70. *kdr‐His* has been reported to coexist with L1014F (*kdr*) in most populations in previous studies, with reported allelic frequencies reaching a maximum of 0.81 in the United States, Turkey, China and Italy (Alzabib et al., 2025; Freeman et al., 2019; Mazzoni et al., 2015; Scott et al., 2013; Taşkın et al., 2011; Wang et al., 2012). In this study, only the pyrethroid resistance mutation *kdr‐His* (allelic frequency: 0.31) was detected in the Nagano population. This suggests a distinct geographical distribution pattern from *kdr* and *super‐kdr* in some areas. Notably, all three populations from Ibaraki Prefecture harboured *super‐kdr* and *kdr‐His* mutations, suggesting possible gene flow among the livestock facilities in this region or similar selection pressures among livestock facilities in this region. Contrastingly, the Nagano population demonstrated only *kdr‐His* (frequency: 0.31), indicating varying selection pressures and introduction pathways in this region. Individuals heterozygous for L1014F and L1014H were detected in only two populations from Chiba Prefecture (0.36–0.47) and Ibaraki Prefecture (0.06) (Table S2). The heterozygote of L1014F and L1014H has been reported in one Italian population with a frequency of 0.33, which is comparable to the result for the Chiba Prefecture population in this study (Mazzoni et al., 2015). However, neither *kdr1B* (T929I + L1014F) (which is considered to confer resistance comparable to *super‐kdr*) nor *skdrD* (D600N + M918T + L1014F) (which is considered to confer stronger resistance than *super‐kdr* (Sun et al., 2017)) were found, suggesting that these mutations have not yet been introduced into Japan.

Notably, the long‐term laboratory‐reared populations revealed distinct resistance profiles, with no pyrethroid resistance mutations observed in the populations from Hokkaido and Fukushima after eight and six years of laboratory rearing, respectively. This observation could be explained by two possibilities: Either these populations completely lacked *kdr* alleles at the time of collection, or, more likely, these alleles were eliminated during laboratory maintenance owing to their associated fitness costs in the absence of insecticide pressure. The *kdr* allele declines in frequency owing to the effects of fitness costs when interbred with the wild‐type allele in insecticide‐free environments (Freeman et al., 2021; Hanai et al., 2018). In the Kagawa population, which had been maintained for four years, pyrethroid resistance mutations were still detected but at reduced frequencies (0.2). This suggests partial elimination of these alleles during the rearing period. Mertz et al. (2023) recently identified the M918V substitution in exon 16c. This alternatively spliced exon is mutually exclusive of exon 16d, which contains the M918T substitution linked to *super‐kdr*. This suggested that exon 16c plays a role in pyrethroid resistance. In the present study, we identified an M918V substitution in a heterozygous individual from the Chiba 2023 population, supporting exon 16c's potential role in pyrethroid resistance. Additionally, M918V was detected in the YPER strain, as previously described (Kasai et al., unpublished data). However, whether this mutation was introduced abroad or arose independently in Japan remains unclear. Elucidating the resistance levels conferred by M918V is necessary for monitoring insecticide resistance.

A comprehensive analysis of the VGSC gene sequence showed a novel I936F mutation in individuals from the Tochigi population (0.03) (Figure S1, File S1). Molecular docking models have indicated the I936 residue's contribution to insect species‐specific sensitivity to DDT (O'Reilly et al., 2006). Moreover, a mutation at a homologous position in *Helicoverpa zea* confers pyrethroid resistance (Hopkins & Pietrantonio, 2010), suggesting that the I936F variant may represent a novel *kdr* mutation in houseflies. In addition to I936F, various other amino acid polymorphisms were noted in all housefly populations collected from the livestock facilities. These polymorphisms are situated away from the relevant region in the domain I of *VGSC*, which is called the second pyrethroid‐binding site (Du et al., 2013; Williamson et al., 1996). Therefore, it is unlikely that these additional polymorphisms contributed to pyrethroid resistance. These results underscore the criticality of analysing complete gene sequences rather than solely focusing on previously identified resistance loci for comprehensive resistance monitoring. Additionally, Exon 28 was found to harbour more polymorphisms compared to other exons, suggesting it is a mutational hotspot. However, since this region is located at the terminus of the VGSC gene, its contribution to pyrethroid resistance is considered low.

This study demonstrated that three OP/carbamate resistance mutations in the *AChE* gene, V260L, G342A/V and F407Y, are widely distributed and occur at high frequencies in housefly populations in Japan. *AChE* resistance mutations were consistently observed at high frequencies (0.5–1.0) across all wild populations, even in those where *kdr* was observed at low frequencies or were not detected at all (Figure 5a–c).

Notably, the three *AChE* resistance mutations identified in our study were all previously detected in the YBOL strain, which was collected in Tokyo in 1989 and subsequently selected as an OP insecticide (Kozaki et al., 2001; Lee et al., 1996). This historical detection suggests that these resistance alleles have persisted in the Japanese housefly populations for more than three decades. Furthermore, although the YBOL strain contained the G342V mutation, this mutation was not observed in the YPER strain (selected with permethrin), which carried the G342A mutation (Kozaki et al., 2009). This observation suggests that the G342V mutation might be less favored than G342A in the absence of a specific OP selection pressure, possibly because of its higher fitness costs (Kozaki et al., 2009).

Genotype frequencies for V260L and G342A/V mutations showed considerable deviations from HWE across all sampled populations. For V260L, the vast majority of individuals were heterozygous for this mutation, with homozygous mutants being remarkably rare, although the mutant allele had a frequency of approximately 0.5 in several wild populations (Figure 5a, Table S1, Figure S2). Similarly, for the G342 locus, we observed a striking pattern in which most individuals were heterozygous for the G342A and G342V alleles, whereas homozygotes for G342V were completely absent from all studied populations. The results for genotype frequencies at the 260 and 342 loci are particularly similar to those reported in 2012 from the Shanghai and Shandong populations in China (Wang et al., 2012), suggesting that this is not a localized evolutionary phenomenon. Conversely, recent reports from studies in Saudi Arabia (Alzabib et al., 2023) and Kazakhstan (Qu et al., 2021) found that the simultaneous presence of heterozygosity for V260L and G342A/V occurred in less than half the individuals, which differed from the results described above.

As reported globally, the G342V mutation has been predominantly detected in the heterozygous state, with homozygosity for G342V being first reported in only a few individuals by 2025 (You et al., 2025). This predominance of heterozygotes is likely explained by functional studies revealing that the G342V mutation decreases the substrate affinity for acetylcholine by more than sixfold compared to other variants (Walsh et al., 2001). Such a substantial decrease in enzymatic function substantially impairs normal neurological processes, thereby imposing a severe fitness cost on individuals homozygous for G342V, even in the absence of insecticide pressure. This functional constraint explains why G342V homozygotes are rare in the field and laboratory populations, despite the clear adaptive advantage of the mutation in insecticide‐resistant heterozygotes.

To evaluate potential gene duplication, read counts of *AChE* obtained by NGS were normalized using read counts of *VGSC*, which was assumed to exist as a single copy in the genome, and the relative RPK was evaluated (Figure 7). In several individuals, *AChE* reads increased more than 1.5 times that of the susceptible strain, with substantial differences observed in 9 out of 15 populations.

This increase in the relative copy number ratio provides clear genomic evidence of AChE gene duplication events in resistant populations. Gene duplication represents a crucial adaptive mechanism, allowing houseflies to maintain one copy that can be mutated to confer insecticide resistance while preserving another copy with normal enzymatic function. This mitigates the fitness costs typically linked to resistance mutations. Although our DNA‐based approach confirmed the presence of gene duplications, determining whether these duplicated genes demonstrate differential expression patterns requires additional RNA‐based studies.

In addition, individuals carrying three different alleles at amino acid position 342 were identified (Figure S3). These individuals were not included in the tally of amino acid mutations or haplotypes of *AChE*. Individuals carrying these three alleles were prevalent in the Ibaraki B and C, Kagoshima and Miyazaki populations, with fewer occurrences in the Fukuoka, Oita and Kagawa populations. These results suggest the presence of gene duplications in the *AChE* locus, including the G342A, G342V and wild‐type alleles.

Similar heterogeneous duplications of the *AChE* locus carrying various resistance alleles have been reported in *Anopheles gambiae*. These duplications have been suggested to decrease the fitness cost linked to resistant *AChE‐1* alleles by maintaining beneficial heterozygosity over time (Assogba et al., 2018; Claret et al., 2024). Thus, we inferred in houseflies that gene duplications containing heterogeneous *AChE* alleles, in addition to point mutations, might similarly contribute to the stabilization of insecticide resistance phenotypes. Figure 6 illustrates the proposed AChE gene structures of individuals suspected to carry heterogeneous gene duplications, along with their observed allele read‐depth ratios.

In Figure 6, notable examples from three wild‐caught individuals are compared with the susceptible strain. Notably, the detection of three different alleles within the same individual strongly suggested gene duplication events. NGS analysis cannot distinguish between identical sequence copies, potentially underestimating the duplication of identical alleles, which is a crucial consideration when evaluating the overall prevalence of AChE gene duplication.

Notably, gene duplication events generally occur at much lower frequencies than point mutations at specific sites. Thus, detecting AChE gene duplications in 12.1% of the analysed individuals suggests a strong selective pressure favouring these structural variations. The presence of three different nucleotide variants (G/C/T) at codon position 342 in these individuals provides compelling evidence of gene duplication because diploid organisms would normally carry a maximum of two variants at any given locus. Distinguishing between normal diploid alleles and duplicated genes becomes more challenging using our current methodology in cases in which only one or two nucleotide variants are detected.

However, we cannot definitively determine the evolutionary sequence: Whether resistance mutations occurred before duplication or whether duplications preceded the acquisition of resistance mutations. This limitation arises because we cannot distinguish between various duplication structures in the wild‐type haplotypes (G/G, GG/G or GG/GG) because NGS analysis cannot directly confirm duplications of identical sequences.

In the Fukushima population, which had been maintained for six years in an insecticide‐free environment, all individuals were heterozygous for the G342A and G342V mutations. Notably, unlike several other populations in this study, the relative copy numbers indicated that the copy number of the AChE gene converged to approximately one in most individuals from this population, providing strong evidence against gene duplication. This result is especially significant because in the absence of gene duplication, consistent heterozygosity at the G342 locus strongly suggests overdominance, a phenomenon in which heterozygotes show higher fitness than homozygotes. Although gene duplication can sometimes create the appearance of heterozygosity in sequencing data (when each duplicated gene carries different homozygous mutations), our copy number analysis ruled out this possibility for the Fukushima population. This pattern of maintained heterozygosity without gene duplication is well documented in insect genetics, especially in studies of *Drosophila melanogaster* (Peng et al., 1991). Additionally, the complete absence of the G342A/G342A and G342V/G342V homozygotes suggests that these genotypes are lethal or severely disadvantageous in this population.

Supporting this interpretation, Figure S4 illustrates the haplotype combinations of the four resistance‐associated mutations based on the sequence data that did not consider gene duplications. This analysis showed that the V260L mutation co‐occurred almost exclusively with G342A. This suggests a strong linkage or functional association between the two. This observation is consistent with previous reports that V260L may mitigate G342A's negative impact on the substrate specificity of AChE (Walsh et al., 2001). Therefore, the substantial deviations from HWE observed for V260L may reflect selection against G342A homozygotes owing to fitness costs, further supporting the role of overdominance at this locus. However, direct experimental confirmation of this overdominance hypothesis is beyond the scope of this study and represents a crucial direction for future research.

Moreover, mutations not previously implicated in resistance (L677V, T666A, I162M, S28Y, S23P, P11S and I9T) were detected in all wild populations. Among these mutations, T666A and P11S were present in the SRS strain. Although I162M's involvement in resistance remains elusive, it is situated near the enzyme substrate recognition site and has been reported in several OP/carbamate‐resistant populations (Kozaki et al., 2001; You et al., 2020). The other identified mutations were situated distant from the enzyme's active site and were thus considered unlikely to contribute to insecticide resistance. These amino acid substitutions situated outside the enzyme's catalytic site likely represent natural genetic polymorphisms between the laboratory‐maintained aabys reference strain and wild houseflies from Japan. Population‐specific mutations were observed in each geographical group, with the Miyazaki B population demonstrating notably higher levels of genetic polymorphisms. The Y226F mutation detected in the Ibaraki A and Chiba populations was identical to that reported at multiple sites in China (Yang et al., 2023). Given its proximity to the active site of the enzyme, its further validation as a novel resistance marker is warranted (Dvir et al., 2010).

As of 2022, insecticides registered for adult fly control in livestock facilities in Japan will include 14 pyrethroids, 11 OPs/carbamates and 4 combinations of pyrethroids and fenitrothion (OP) (JVPA, 2004). The continued reliance on these insecticides raises concerns about the potential for the accelerated development and spread of resistance in housefly populations. Thus, optimizing the use of available insecticides through careful rotation strategies while also exploring complementary non‐chemical control methods where feasible is necessary to manage the development of resistance within the practical constraints of livestock environments.

This study represents the first comprehensive analysis of insecticide resistance‐associated mutations in Japanese housefly populations using NGS‐based probe capture methodology. Our results revealed a widespread distribution of *VGSC*‐ and *AChE* resistance mutations across livestock facilities, with distinct regional patterns and evidence of evolutionary adaptation. Notably, we provide the first genomic evidence of AChE gene duplication based on copy number analysis, which likely serves as an adaptive mechanism to mitigate the fitness costs linked to resistance mutations. The discovery of heterogeneous duplications containing different resistance alleles, along with the evidence of overdominance in laboratory‐maintained populations, underscores the complex evolutionary strategies used by houseflies under insecticide selection pressure.

While this study focused on comprehensive genetic characterization of resistance mutations, we acknowledge that integrating phenotypic bioassay data would provide a more complete understanding of resistance levels in these populations. Establishing correlations between resistance allele frequencies and actual insecticide susceptibility is essential for practical resistance management. However, such integrated analyses were not feasible in the current study, as our molecular analyses primarily utilized ethanol‐preserved specimens collected over multiple years. Future investigations combining both genotypic and phenotypic approaches, using live specimens with well‐characterized genetic backgrounds, will be necessary to establish robust genotype–phenotype relationships and their implications for resistance management strategies in Japanese livestock facilities.

Future structural validation and comprehensive characterization of the AChE gene duplication architecture using advanced technologies, such as long‐read sequencing, will be indispensable for a complete elucidation of the complex evolutionary dynamics of insecticide resistance. Such insights will improve our fundamental understanding of adaptation mechanisms in response to anthropogenic selective pressures as well as inform the development of more sustainable and effective resistance management strategies in livestock environments worldwide.

## AUTHOR CONTRIBUTIONS

**Mikie Nakagawa:** Data curation; formal analysis; investigation; visualization; writing – original draft. **Kentaro Itokawa:** Methodology; software; validation; writing – review and editing. **Nozomi Uemura:** Validation. **Yasuhiro Tomioka:** Investigation; resources. **Tsutomu Tanikawa:** Investigation; resources. **Kyo Itoyama:** Supervision. **Shinji Kasai:** Conceptualization; funding acquisition; writing – review and editing. **Osamu Komagata:** Project administration; resources; software; supervision; validation; writing – review and editing.

## CONFLICT OF INTEREST STATEMENT

The authors declare no conflicts of interest.

## Supporting information


**Figure S1.** Heatmap of amino acid substitutions in the voltage‐gated sodium channel gene in houseflies from livestock barns.
**Figure S2.** Heatmap of amino acid substitutions in the acetylcholinesterace in houseflies from livestock barns.
**Figure S3.** Allelic distribution and depth at the G342A/V site of the acetylcholinesterase gene allelic depths at the G342A/V substitution site in the acetylcholinesterase gene are shown for each individual. Each stacked bar represents the read‐based allele composition from next‐generation sequencing (NGS), with blue indicating the G342A allele (A variant), red the G342V allele (V variant) and grey the wild‐type G allele. Numbers within each segment indicate the allele depth for that specific base. Data from these individuals are not used in the figures and tables for the tabulation of other *AChE* amino acid mutations.
**Figure S4.** Estimated haplotype combinations carried by individuals in each population, shown as stacked bar plots. Each bar represents the haplotype composition of one population. Grey indicates the wild type, light green indicates V260L, yellow indicates V260L + G342A, light blue indicates G342V + F407Y and red indicates V260L + G342A + F407Y.
**Table S1.** Genotype frequencies of amino acids 162, 260, 342 and 407 of acetylcholinesterase in houseflies from livestock barns.
**Table S2.** Genotype frequencies of Amino Acids 918 and 1014 of voltage‐gated sodium channel in houseflies from livestock barns.

**File S1.** Amino acid number.

## Data Availability

All sequencing data generated in this study have been deposited in the DNA Data Bank of Japan (DDBJ) Sequence Read Archive (https://www.ncbi.nlm.nih.gov/ https://ddbj.nig.ac.jp/search/) under the BioProject accession PRJDB35479 (Nakagawa et al, 2025). Individual sample data are available under the BioSample accessions (SAMD01578869‐SAMD01579108) linked to this BioProject, and raw sequencing data (paired‐end FASTQ files) can be accessed through the DDBJ Sequence Read Archive (DRA) under accession numbers (DRR701119‐DRR701358) (Nakagawa et al., 2025) linked to this BioProject. The Supplementary material provides additional details regarding the deposited data.
